# Potential Use of Common Administration of Emulsion for Parenteral Nutrition and Vinpocetine: Compatibility Study and Prospect

**DOI:** 10.3390/metabo14080439

**Published:** 2024-08-07

**Authors:** Szymon Tomczak, Kornelia Kaszuba, Jagoda Szkudlarek, Ludwika Piwowarczyk, Anna Jelińska

**Affiliations:** Department of Pharmaceutical Chemistry, Poznan University of Medical Sciences, 3 Rokietnicka, 60-806 Poznań, Polandlpiwowarczyk@ump.edu.pl (L.P.);

**Keywords:** compatibility, vinpocetine, supportive drugs, parenteral nutrition, interaction, PFAT5

## Abstract

Vinpocetine (VP) is distributed after oral and intravenous administration, and its uptake in the thalamus, basal ganglia, and visual cortex. Due to poor bioavailability (~7%) and marked first-pass effect (~75%), including a short half-life (2–3 h), oral administration of VP is limited. It requires frequent administration of the drug to obtain a therapeutic effect. Attempts to overcome these difficulties include the use of new drug delivery systems and/or alternative routes of drug administration. One possibility is the common administration of lipid emulsion and drug using the same catheter. However, this procedure is not recommended due to potential interaction and lack of safety data. For this purpose, we checked the compatibility of VP solutions with eight commercially available parenteral nutrition admixtures, i.e., Lipoflex special, Omegaflex special, Lipoflex peri, Omegaflex peri, Kabiven, SmofKabiven, Kabiven Peripheral, and Olimel Peri N4E. Coadministration is only possible if the stability of the drug and the lipid emulsion is confirmed. The available data are scarce and only concern the incompatibility of VP with ibuprofen. Compatibility tests were carried out in simulated administration through a Y-site connector using clinical flow rates. The stability of the drug and lipid emulsion was assessed by visual inspection and measurement of pH, osmolality, particle size as mean droplet diameter (MDD) and percentage of lipids residing in globules larger than 5 µm (PFAT5), zeta potential, polydispersity index, and lipid-free parenteral nutrition admixture(PNA) turbidity. The results of the compatibility of VP with eight commercial PN admixtures showed that all lipid emulsions show different signs of destabilization. In the studied samples, particles larger than 1000 nm, a significant increase in MDD, zeta potential, and loss of homogeneity visible as an increase in the polydispersity index were observed. Most of the samples had PFAT5 above the USP limit (0.05%). Taking into account the obtained data, VP should not be administered with the studied lipid emulsions for parenteral nutrition.

## 1. Introduction

Neurodegeneration, stroke, and other brain disorders are common causes of hospitalization and deterioration of a patient’s condition, affecting both prognosis and life quality [1]. In these cases, nootropic drugs like vinpocetine (VP) are often used. VP is a semi-synthetic derivative of vincamine, a secondary metabolite derived from *Vinca minor* L. belonging to the *Apocynaceae* plant family [2].

VP has many indications in neurodegeneration disorders, such as treatment of acute cerebral ischemic states, including paroxysmal, transient ischemic attack (TIA), and ischemic stroke, as well as post-stroke conditions when parenteral treatment is necessary [3,4]. VP is also indicated for treating chronic circulatory disorders in the choroid and retina (e.g., thrombosis, occlusion of the central retinal artery or vein). The mechanism of action is complex and includes phosphodiesterase inhibitor(PDE) inhibition. Due to its effect on improving cerebral circulation, it is used as a dietary supplement in some countries, but in some, its use is limited only to physician prescriptions. Due to its wide range of effects, VP is often used in hospital wards, constituting one of the components of drug regimens used in patients who struggle with many health problems and require comprehensive posology. These indications place VP as an important supporting drug during treatment and convalescence in patients undergoing parenteral nutrition.

Nutritional support is an important element of the proper treatment process. A poor nutritional status of a patient negatively affects their treatment and convalescence [5]. Clinical nutrition is an essential aspect of treatment as a drug administration. Clinical nutrition may be determined depending on patients’ needs, contraindications, or route of administration. Enteral nutrition may be oral nutrition supplements (ONS) or more invasive, using percutaneous endoscopic gastrostomy (PEG) or jejunostomy [6]. Parenteral nutrition is usually implemented only when enteral supply is insufficient or impossible. Hence, parenteral nutrition, as a practice with the most significant risk, is used only in a narrow group of patients where enteral nutrition is impossible [7]. An essential aspect of the safety of therapy is to pay attention to possible drug interactions.

Drug administration directly by tubes is routine practice in patients receiving enteral nutrition. Nevertheless, it is worth remembering that not every oral pharmaceutical form can be administered this way. Modified-release medication or enteric-coated crushed tablets administered through a gastric tube will not have the proper therapeutic effect. Modified-release tablets may release the active substance faster and thus cause peak concentration in plasma, and a destroyed enteric-coated drug coating, e.g., omeprazole, will cause a lack of therapeutic effect due to the deactivation of the API in gastric acid. Other important interactions are interaction with food (i.e., VP bioavailability can be influenced by food intake, particularly a high-lipid meal that causes changes in serum concentration [2]), tube obstruction, drug absorption on medical devices, and drug–drug interaction when administered together [8]. However, taking several drugs together increases the risk of interactions in the pharmaceutical, pharmacodynamic, or pharmacokinetic phase. Precipitation, color change, reduction in drug concentration, or inactivation are drugs’ most common pharmaceutical phenomena. Due to poor bioavailability and marked first-pass effect (~75%), oral administration of VP is limited and requires frequent drug administration to obtain a therapeutic effect [9]. Using lipid emulsion in polytherapy increases the incidence of potential interactions during administering parenteral fluids. Working with emulsions must be considered as they are thermodynamically unstable systems and may be separated into two phases. The lipid emulsion, used as nutritional support or as a single source of nutrient supply, is often introduced to malnourished, unconscious patients who cannot take food enterally [7], mainly in critically ill or cancer patients.

Emulsions are biphasic liquid systems where one liquid phase, known as the internal or dispersed phase, is dispersed as small droplets through the second fluid phase, known as the external or continuous phase. Lipid emulsion is a promising supporting drug delivery vehicle [10]; compatibility was proved, for example, with linezolid or levetiracetam [11,12]. Drugs such as propofol, doxorubicin, daunorubicin, and amphotericin B have already been introduced in these forms into clinical practice [13,14]. Many factors influence the stability of the lipid emulsion, such as ion concentration, the presence of amino acids, solution pH, and temperature. Hence, it is important to constantly update the current state of knowledge and further develop science about potential drug–admixture interactions. Thus, we decided to check the compatibility of VP solutions with eight commercially available parenteral nutrition admixtures (PNAs). A physicochemical evaluation was performed using simulated co-administration through a Y-site.

Compatibility assessment was carried out through a series of validated measurements, i.e., visual inspection, pH measurement, osmolality, particle size expressed as mean droplet diameter (MDD) using the dynamic light scattering(DLS) method, percentage of lipids residing in globules larger than 5 µm (PFAT5) obtained using the light obscuration method, non-lipid fraction turbidity, zeta potential, and polydispersity index. To the best of our knowledge, no study has extensively explored the potential interactions of such combinations.

## 2. Materials and Methods

### 2.1. Materials: Vinpocetine Solutions and Parenteral Nutrition Admixtures

VP was used under the brand name Cavinton^®^ as a concentrate for infusion at 5 mg/mL (Gedeon Richter, Budapest, Hungary) (EXP 03.2024, LOT A93002). According to the Summary of Product Characteristics (SmPC), Cavinton is administered in the form of dilution in 500 mL of 5% glucose solution (5Gl) or 0.9% physiological saline solution (NS). The maximum administration rate is 80 drops per minute (V_max_ = 240 mL/h), and the entire drug solution should be administered to the patient within 3 h of preparation (V_min_ = 167 mL/h). The average treatment duration is 10–14 days and the usual daily dose for a patient weighing 70 kg is 50 mg/day (5 ampoules in 500 mL of solution for infusion).

The eight commercially available PNAs were analyzed in the study. The PNAs differ in electrolyte content and injectable lipid emulsion (ILE) source. Lipoflex special and Lipoflex peri are made of Lipofundin MCT/LCT^®^ (B. Braun, Melsungen, Germany), which contains refined soybean oil and medium-chain triglycerides. Omegaflex special and Omegaflex peri are enriched with ω-3-acid triglycerides, which are components of Lipidem^®^(B. Braun, Melsungen, Germany). Whereas Kabiven and Kabiven Peripheral PNAs are based on Intralipid^®^ (Frasenius Kabi AB, Uppsala, Sweden) 20%, which is made up of soybean oil, egg yolk phospholipids, and glycerin, SmofKabiven(Frasenius Kabi AB, Uppsala, Sweden) is based on SMOFlipid 20% (Frasenius Kabi AB, Uppsala, Sweden), which consists of a mixture of soybean oil, medium-chain triglycerides, olive oil, and fish oil (rich in ω-3 fatty acids). Olimel Peri N4E (Baxter, Warsaw, Poland) contains refined olive oil and refined soybean oil. The summary of PNA compositions is presented in Table 1.

After activation, each PNA was supplemented with vitamins and trace elements. Lipoflex special, Lipoflex peri, Omegaflex special, and Omegaflex peri were supplemented with Viantan as a vitamin source and Tracutil (both manufactured by B. Braun Melsungen AG, Melsungen, Germany) as trace elements. One ampoule corresponds to the daily requirement. The Soluvit N and Vitalipid N Adult (both manufactured by Fresenius Kabi AB, Uppsala, Sweden) were added to Kabiven, Kabiven Peripheral, and SmofKabiven. The source of trace elements was one ampoule of Addamel N (Fresenius Kabi AB, Uppsala, Sweden). To Olimel Peri N4E was added Cernevit (Baxter, Warsaw, Poland) and one ampoule of Tracutil. Such a wide and varied selection of the PNA composition is intended to review potential drug: admixture interactions.

### 2.2. Methods: Compatibility Evaluation

The VP solution and PNAs ratios were calculated based on maximum and minimum flows. The two extreme ratios were used in the study. The third ratio, 5:5, is often out of clinical use but was added to compare the result and is often used by previous authors [15,16,17,18,19]. The drug:PNA ratio mimics a clinical scenario when the drug has direct contact with other fluids in a common line of Y-site catheter. The study was conducted in a static way, i.e., the drug solution and PNA were mixed in a plastic tube in a calculated ratio and immediately analyzed at the first endpoint (t_0h_) and after four hours (t_4h_). This procedure allows you to capture interactions that have progressed over time. The samples were stored at 21 ± 2 °C, with light access during the test. The choice of the second endpoint (t_4h_) as the final measuring point is consistent with the methodology presented by other researchers [20,21,22,23]. The time of 4 h is beyond clinical justification because the contact between the drug and the mixture is much shorter. Still, it allows for detecting certain signs of incompatibility that develop over time, e.g., precipitation of calcium or phosphates [24].

Due to the very complex structure and the multitude of potential interactions, it is necessary to carry out various measurements analyzing individual parameters of the complex matrix. Each measurement was performed in triplicate (*n* = 3) and expressed as a mean with a standard deviation

#### 2.2.1. Visual Control

The visual control was performed based on the procedure described in European Pharmacopoeia [25]. Two independent investigators analyzed the samples. This is the first stage of the PNA analysis. Its purpose is to capture with the unaided eye sign of destabilization, i.e., color change, delamination, sedimentation, gas formation, or other aging processes.

#### 2.2.2. The pH Measurement

Before starting the measurements, the instrument (Mettler Toledo Seven Compact pH/Ion S 220 pH meter, Mettler Toledo, Columbus, OH, USA) was calibrated using three standards. The pH meter electrode was placed directly in the investigated sample. The electrode was rinsed with distilled water between each measurement.

#### 2.2.3. Osmolality Measurement

Osmolality was measured on the osmometer 800CLG (TridentMed, Warsaw, Poland). A volume of 100.0 µL of the sample was pipetted to the Osmo-Krio vials. The instrument was calibrated before starting the series of measurements with Osmometer Calibration Solution 800 cL, 0 mOsm/kg H_2_O, Cat. Yes. 800.02 (TridentMed, Warsaw, Poland). This measurement was based on the freezing point depression method. One mole of a non-dissociating substance dissolved in 1 kg of water decreases the freezing point of the resultant solution by 1.86 °C. One Osm corresponds to 1 mole of a chemical compound exhibiting osmotic activity dissolved in 1 kg of water.

#### 2.2.4. Measurement of Turbidity

Turbidity measurement was performed on a TU52000 Laboratory Laser Turbidimeter (Hach Company, Loveland, CO, USA). Method specification requires a transparent sample. Instead of three, only two chambers of RTU bags were activated. Then, water for injection was added to an equal volume of lipid emulsion and supplemented with trace elements (see point 2.1). The drug:lipid-free PNA ratios remain the same. Turbidity was measured using 10 mL of sample in special cuvettes placed in a turbidimeter cell.

#### 2.2.5. Measurement of Droplet Size, PDI, and Zeta Potential

The solutions, prepared by mixing 1 mL of sample with 9 mL of distilled water, were transferred to the U-shape cuvette. All three parameters were analyzed in parallel with the ZetaSizer Nano ZS apparatus (Malvern Instruments Ltd., Malver, UK). The size was measured using the DLS method and expressed as MDD mean particle size (based on their diameter in nm) and polydispersity index (PDI). The zeta potential was expressed in mV.

#### 2.2.6. Particle Size Measurements (LO)

The lipid emulsion sample was diluted in a ratio of 1:2000 using water for injection and then analyzed by the PAMAS particle counter (PAMAS Partikelmess- und Analysesysteme GmbH, Rutesheim, Germany). This instrument utilizes the light obscuration method, which allows the counting and sizing of subvisible particles on a specific level. The percentage of lipids residing in globules larger than 5 µm (PFAT5) was calculated based on previous descriptions [26].

#### 2.2.7. Statistical Analysis

The results were expressed as averages of 3 separate measurements with standard deviation. To statistically evaluate the obtained results, one-way ANOVA followed by Tukey’s post hoc test was applied (Statistica 12 software, StatSoft Polska, Cracow, Poland). A value of α = 0.05 was considered statistically significant [27].

## 3. Results

### 3.1. Characterization of Total Parenteral Nutrition Admixtures without Drug Solutions

The PNAs were activated according to the manufacturer’s procedure, and all passed the visual control. Their pH ranged from 5.48 up to 6.40 for Omegaflex peri and Olimel Peri N4E, respectively. The route of administration defined osmolality. The osmolality of PNA for central access was from 1153 ± 8 to 2008 ± 6 (Kabiven and Lipoflex special, respectively), and the osmolality of PNAs for peripheral access was lower than 1000 mOsm (795 ± 2-Kabiven Peripheral and 921 ± 4 Omegaflex peri). When it comes to particle size characterization, samples showed droplet sizes well below the limit of acceptance criteria of stable PNA emulsions. The size was in the narrow range in all lipid emulsions, with a minimum value of 243.4 ± 3.7 and a maximum of 281.7 ± 0.3 (SmofKabiven and Kabiven, respectively). These differences show the impact of this parameter on lipid sources. Both PNAs were produced by Fresenius Kabi but differ in an injectable lipid emulsion. Kabiven contains soybean oil with the lowest value, and SmofKabiven contains a mixture of soybean oil, medium-chain triglycerides, olive oil, and fish oil, which affects the biggest lipid droplet. Zeta potential was negative for all PNAs and lower for the peripheral route of administration. The lipid-free samples were clear, and turbidity was low, below 0.45 NTU. Table 2 summarizes the properties of fluids used in the study: parenteral fluids, drug solutions, and PNA without drug addition.

### 3.2. Compatibility Test Assessment

The eight commercially available, ready-to-use PNAs were used in the compatibility study with two VP solutions. Preparing a VP solution in a 5% glucose and saline solution lowers their pH value. All tested samples were homogeneous, free of crystals, gas, and other signs of destabilization, and unaffected by time (0–4 h).

Adding an acidifying drug solution lowers the pH value of all PNAs, but the buffer capacity (electrolytes, amino acids) remains at a safe level. No significant changes (*p* < 0.05) were observed during the evaluation period. A maximum increase of 0.07 was observed for the Lipoflex special sample mixed with VP in NS in a ratio of 8:2. A drug solution with low osmolality causes a significant decrease in these parameters in mixed samples, most of them below 1000 mOsm, which is due to the high content of drug solution in the mixture. After 4 h, no significant changes were observed, but the largest for the Kabiven admixture was an increase in RSD of 2.4%, which is significantly below the acceptability criterion (RSD > 5%). The analyzed turbidity of lipid-free admixtures with the drug did not show changes greater than +0.5 NTU during the test, which indicates the lack of precipitation of the drug or insoluble salts [28]. The results of pH, osmolality, and turbidity are presented in Table 3.

**Figure 1:**
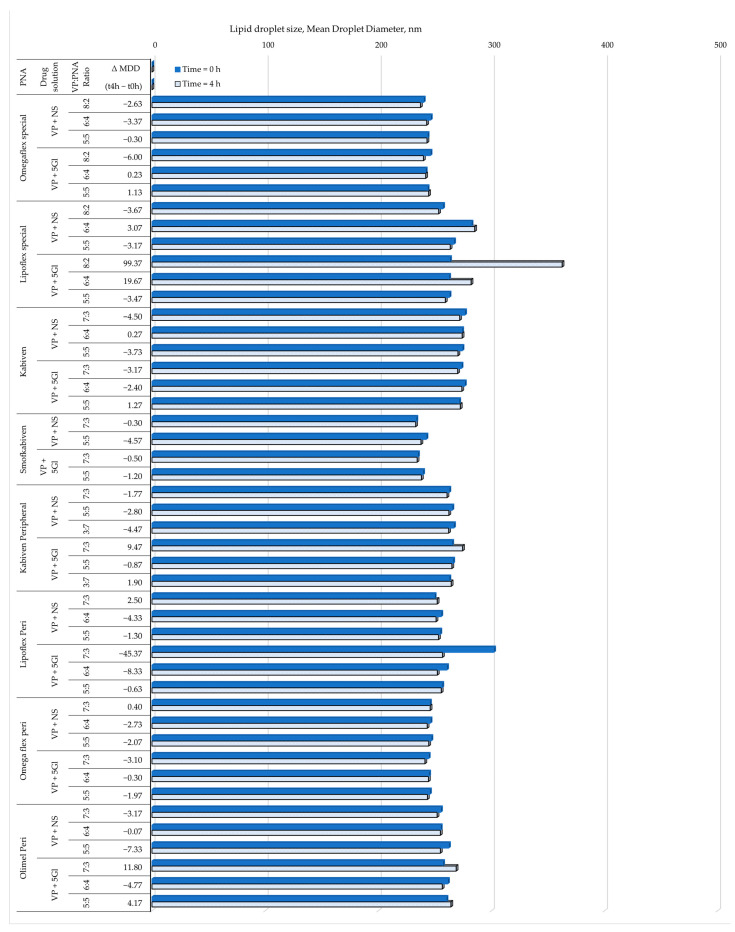
The results of particle size measurements (mean droplet diameter).

Mixing the drug solution did not significantly affect the average size of the lipid emulsion droplets, which at two measurement end-points were within the USP limit, i.e., below 500 nm [29]. However, mixing the VP glucose solution with Omegaflex special resulted in a significant increase in MDD by as much as 99.4 nm (38%), and reducing the share of the drug solution in glucose to 6:4 resulted in an increase in MDD by 19.7 nm (the second largest observed increase) (Figure 1). The presence of the second fraction of particles larger than 1000 nm was noted in all PNAs, regardless of the type of lipid emulsion. The light obscuration method used for particle size measurements confirmed the above data. All samples containing a second fraction of particles larger than 1000 nm were also outside the limit for PFAT5. However, the LO method allows the capturing of more incompatible samples. A total of 29 out of the 46 samples had PFAT5 larger than the criteria of 0.05% [29].

**Figure 2:**
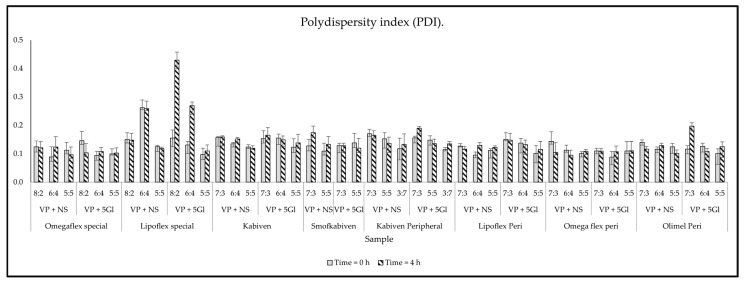
The results of polydispersity index (PDI).

**Figure 3:**
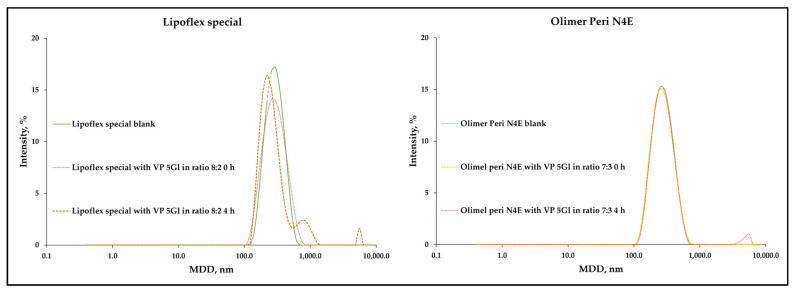
Lipid droplet size intensity for Lipoflex special sample (**left**) and Olimel Peri N4E (**right**).

**Figure 4:**
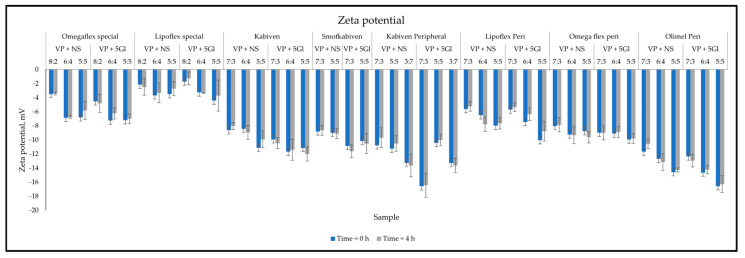
The results of zeta potential measurements.

The loss of sample homogeneity resulting from the significant increase of lipid droplets was observed in the change in PDI values after 4 h, which reached values of 0.429 ± 0.031 and 0.269 ± 0.031 (Figure 2). This is indicative of a wide-size distribution, which is desirable in connection with emulsion instability. Lack of homogeneity and aggregation of lipid droplets are visible in the intensity graph by shifting the graph to the right, tailing it (Figure 3, left side), or by visible additional intensity peaks (Figure 3, right side). Changes related to the destabilization of the lipid emulsion were also observed in the zeta potential assessment. While the storage time did not have a significant effect, the addition of drug solutions shifted it to the value of 0 mV, i.e., the point at which the forces of the dispersion system disappear. The greatest change to 0 mV value was also observed for Lipoflex special and VP 5Gl in a ratio of 8:2 (+6.44 mV) (Figure 4).

## 4. Discussion

The VP is distributed after both oral and intravenous administration, and its uptake in the thalamus, basal ganglia, and visual cortex [3]. Due to poor bioavailability (~7%) and marked first-pass effect (~75%), including a short half-life (2–3 h), oral administration of VP is limited and requires frequent administration of the drug to obtain a therapeutic effect [9]. An attempt to overcome these difficulties seems to be the use of nanoformulations as drug delivery systems and/or alternative routes of drug administration. Congcong Lin et al. proposed the development of a new oral delivery system for VP based on the preparation of vinpocetine–cyclodextrin–tartaric acid complexes and loading them into lipid nanocarriers (NLCs) to improve bioavailability. The improvement was achieved for the nanocomplex in the range of 592% compared with vinpocetine suspension and 92% higher than vinpocetine–NLC [30]. Interestingly, Shuangshuang Song et al. reported that proniosome formulation with vinpocetine showed significantly improved bioavailability compared to vinpocetine suspension in vivo study in rabbits. The incorporation of vinpocetine into niosomes improved the absorption of the drug. The area under the concentration versus time curve AUC (0−∞) F2 and F3 was approximately 4.0 and 4.9 times higher, respectively, than for the free vinpocetine suspension. Those results may prove that proniosomes improve the absorption after oral administration of poorly water-soluble drugs [31]. Transdermal drug delivery is an important alternative way for substances that are problematic when administered orally due to their low bioavailability and poor solubility [32]. Recent research focuses on transdermal administration as an alternative approach also for infusion. Praveen K Srivastava et al. demonstrated a liposomal fast-dissolving microneedle patch of vinpocetine. An in vivo study in rats showed a three-fold increase in relative bioavailability compared to oral administration [33]. Moreover, Sumaia Abdulbari Ahmed Ali Hard et al. proposed intranasal administration of vinpocetine-loaded chitosan nanoparticles (VP-CH-NP) to minimize systemic exposure. Intranasal administration of the in situ gel in an in vivo study in rats demonstrated a two-fold increase in C_max_ (*p* < 0.05) and AUC0-t (*p* < 0.05) in the brain in comparison to oral administration. Moreover, histopathological examination of the nasal mucosa did not show any cytotoxic effect of the tested nanoformulation [34]. In another study, Ahmed et al. made lipid-based nanocarriers loaded with vinpocetine in the in situ gel system (ISG), among which nanomicelles VPN-D-α-tocopherol and polyethylene glycol 1000 (TPGS) were characterized by the smallest particle size (13 ± 2 nm) and the highest encapsulation efficiency (100%). In an in vivo study on rats, vinpocetine-loaded TPGS-ISG micelles showed a higher drug concentration in the brain tissue in intranasal administration compared to ISG micelles and a commercial drug [35].

The presented new delivery solution states the question of whether PNA can be used as a drug vehicle. The utilization of lipid emulsion as a supporting drug administration is possible and used in clinical practice. It increases bioavailability, improves the pharmacokinetic parameters of the drug, and reduces costs [10,13]. However, the co-administration of two drugs through a common venous access may be risky to the patient’s health and life. Interactions resulting from such a common supply have been reported, i.e., ondansetron destabilized lipid emulsion with oiling out [15], precipitation of ciprofloxacin [17], the occurrence of larger lipid droplets after mixing with vancomycin [36], sodium valproate [37], or ibuprofen [38]. The supply of an admixture containing larger particles is a serious threat to life resulting from the blockage of small blood vessels in the brain, liver, or eye [39,40,41]. Due to the high risk of this procedure, it is necessary to carefully check whether it is possible. The current regulations clearly state that the addition of a drug to a PNA is only possible if the physicochemical compatibility and stability, as well as clinical effectiveness data, confirm the expected therapeutic effect of the drug [42]. If available data are insufficient, each fluid has to be administered via a separate catheter or flush drain between administrations. So far, there are no data describing the compatibility of VP with PNAs; scarce and limited data are available about the incompatibility of VP with some drugs, such as ibuprofen in doses of 400 and 600 mg [38]. This lack of specific knowledge highlights the importance of expanding research and filling the knowledge gap, especially concerning different types of PNA. Therefore, this study aims to provide evidence on whether coadministration is possible. To assess compatibility, it is crucial to establish acceptance criteria. Thus far, there are no external guidelines or procedures for testing the compatibility of PNA with drugs, so it is important to test many parameters of the PNAs.

The visual control aims to determine at the first stage whether the admixture shows no signs of aging or dangerous interactions visible by the unaided eye. The pH measurement provides information about ongoing aging processes, such as the rancidity of lipids, as a result of which free fatty acids are released, lowering the pH of the admixture. The pH value of PNA depends on many ingredients, including glucose increases acidity, while amino acids and electrolytes create a buffer capacity that maintains a constant pH of the admixture. Too low or high pH destabilizes the stability of the lipid emulsion. Most drug substances are weak acids or bases, so their solubility depends on the pH value. If there is a large difference in the solubility of the dissociated salt form to the free undissociated base or acid, even a small change in the pH of the solution may cause interaction, which is very dangerous. There may be a parallel influence: the patient is subjected to ineffective therapy and receives subtherapeutic doses, and in addition, too large particles that enter the venous system may cause an embolism. Cases of drug incompatibility have been reported when combined with a low-pH PNA (ampicillin and fosphenytoin precipitate when mixed with Numeta) [43]. It was assumed that a value of 5.5 or lower increases the sensitivity to the breakdown of the lipid emulsion [44]. Of the tested mixtures, only Olimel Peri N4E had a higher pH in the range of 6.01–6.26. However, pH changes greater than 0.2 units during analysis (4 h) were considered as an indication of incompatibility. The tested samples met this requirement because the largest change was only +0.07. A significant decrease in sample osmolality over time may also be related to drug precipitation. As a result of the tests, the observed changes in osmolality ranged from −1.2 to 2.4% and were lower than the accepted limit of RSD < 5%. Turbidity measurement is another analytical method to ensure the reliability and detection of sediment in solution. Turbidity, a measure of the opacity of a liquid, is considered and simple indicator of quality; among others, injectable drugs or drinking water whose value is less than 1 NTU (nephelometric turbidity unit). According to the WHO, “crystal clear” water has a turbidity of <1 NTU; at 4 NTU and above the water becomes noticeably cloudy. The larger the amount that allows access to the sample, the greater the scattering of the incident rays and the greater the turbidity [45]. An increase in turbidity greater than 0.5 NTU during analysis indicates incompatibility. Measurements so far do not provide data on the precipitation of VP when mixed with PNA. The maximum increase of turbidity was captured for the Kabiven Peripheral sample mixed in the ratio 3:7 with VP in 5% glucose and was only +0.115 NTU. However, the key parameter remains the size of the lipid emulsion particles measured as MDD, as well as the presence of the so-called second fraction of particles larger than 1000 nm. The USP defines the upper limit for MDD as 500 nm [29]. Throughout the study, no sample exceeded the USP limit, although a significant increase of as much as 99 nm in MDD was observed for the VP in the 5% glucose sample with Lipoflex special in a ratio of 8:2. Despite the average particle size being within the limit, the presence of a second fraction was observed for most of the PNAs mixed with VP. A total of 29 of the 46 investigated samples were above the PFAT5 limit (>0.05%), which also proved aggregation of lipid droplets and incompatibilities. These changes were also well observed through the increase in PDI. These results prove that the mixing of VP and PNA does not affect the precipitation of the drug, but it does negatively affect the stability of the lipid emulsion, which aggregates into larger particles and thus loses its homogeneity. The zeta potential measurements also capture these changes, and aggregation of lipid droplets leads to a decrease in interaction forces, and we observe the changes zeta potential values, which drives further fusion of particles [46]. A summary of the compatibility study is presented in Table 4.

## 5. Conclusions

The compatibility study of VP solution in 0.9% normal saline or 5% glucose with eight parenteral nutrition admixtures (Lipoflex special, Omegaflex special, Lipoflex peri, Omegaflex peri, Kabiven, SmofKabiven, Kabiven Peripheral, and Olimel Peri N4E) proved the occurrence of interactions. Despite the lack of precipitation of the drug, the signs of breakdown of the lipid emulsion were observed, i.e., an increase in PDI and zeta potential, or the presence of the second fraction of particles larger than 1000 nm for most PNAs samples, which disqualifies the tested lipid emulsions as a base for the administration of vinpocetine parenteral solutions.

## Figures and Tables

**Table 1:** Composition and energy value of the tested PNA per 1000 mL.

Ingredient	Omegaflex Special	Omegaflex Peri	Lipoflex Special	Lipoflex Peri	SmofKabiven	Kabiven	Kabiven Peripheral	Olimel Peri N4E
ILE	Lipidem^®^	Lipidem^®^	Lipofundin^®^	Lipofundin^®^	SMOFlipid^®^	Intralipid^®^	Intralipid^®^	ClinOleic^®^
Lipids [g]	40	40	40	40	38	39	35	30
MCT [g]	20	20	20	20	11	-	-	-
Soybean oil [g]	16	16	20	20	11	39	35	6
Fish oil [g]	-	-	-	-	5.7	-	-	-
Olive oil [g]	-	-	-	-	9.6	-	-	24
ω-3 fatty acids [g]	4	4	-	-	-	-	-	-
Glucose [g]	144	64	144	64	127	97	67	75
Amino acids [g]	56	32	56	32	50.8	33.1	23.6	25.3
Nitrogen [g]	8	4.6	8	4.6	8.1	5.3	3.8	4
Total energy [kcal]	1184	764	1184	764	1084	909	695	700

ILE—injectable lipid emulsion; MCT—medium-chain triglycerides.

**Table 2:** Summary of parameters of used fluids.

Sample	pH ± SD	Osmolality ± SD (mOsm/kgH_2_O)	Turbidity ± SD (NTU)	ZP ± SD (mV)	PDI ± SD	MDD ± SD (nm)
parenteral fluids						
0.9% NaCl	6.60 ± 0.01	289 ± 1	0.093 ± 0.007	N/A	N/A	N/A
5% glucose	4.85 ± 0.01	291 ± 1	0.160 ± 0.004	N/A	N/A	N/A
drug solution						
VP+ 0.9% NaCl	3.48 ± 0.02	296 ± 1	0.168 ± 0.012	N/A	N/A	N/A
VP + 5% glucose	3.71 ± 0.04	301 ± 2	0.174 ± 0.003	N/A	N/A	N/A
parenteral nutrition admixtures						
Omegaflex special	5.57 ± 0.01	1932 ± 6	0.186 ± 0.004	−9.5 ± 0.2	0.111 ± 0.020	251.2 ± 1.8
Omegaflex peri	5.48 ± 0.00	921 ± 4	0.133 ± 0.015	−14.3 ± 0.5	0.095 ± 0.014	249.9 ± 5.3
Lipoflex special	5.51 ± 0.01	2008 ± 6	0.134 ± 0.002	−8.35 ± 0.6	0.102 ± 0.024	267.3 ± 3.8
Lipoflex peri	5.74 ± 0.00	909 ± 1	0.121 ± 0.004	−11.2 ± 0.1	0.116 ± 0.008	256.5 ± 1.7
SmofKabiven	5.47 ± 0.00	1582 ± 14	0.220 ± 0.002	−11.6 ± 0.3	0.119 ± 0.013	243.4 ± 3.7
Kabiven	5.51 ± 0.01	1153 ± 8	0.441 ± 0.014	−10.8 ± 0.5	0.133 ± 0.028	281.7 ± 0.3
Kabiven Peripheral	5.58 ± 0.00	795 ± 2	0.223 ± 0.002	−15.0 ± 0.5	0.130 ± 0.023	267.2 ± 3.0
Olimel Peri N4E	6.40 ± 0.00	850 ± 1	0.209 ± 0.021	−17.2 ± 0.4	0.113 ± 0.018	256.5 ± 1.5

VP—vinpocetine; NTU—nephelometric turbidity unit; N/A—not applicable; PDI—polydispersity index; ZP—zeta potential; MDD—mean droplet diameter.

**Table 3:** Results of pH, osmolality, and turbidity measurements from compatibility study.

PNA	Drug Solution	VP:PNA Ratio	pH			Osmolality, mOsm/kg H_2_O			Turbidity, NTU		
			Average ± SD		Δ (t_4h_ − t_0h_)	Average ± SD		Δ%	Average ± SD		Δ (t_4h_ − t_0h_)
			t_0h_	t_4h_		t_0h_	t_4h_		t_0h_	t_4h_	
Omegaflex special	VP + NS	8:2	5.29 ± 0.00	5.23 ± 0.01	−0.06	559.5 ± 2.1	559.0 ± 4.2	−0.5	0.112 ± 0.002	0.112 ± 0.002	−0.109
		6:4	5.46 ± 0.00	5.41 ± 0.01	−0.05	857.5 ± 0.7	860.5 ± 0.7	3.0	0.228 ± 0.004	0.145 ± 0.040	−0.083
		5:5	5.48 ± 0.00	5.47 ± 0.01	−0.01	998 ± 0.0	998.5 ± 0.7	0.5	0.198 ± 0.006	0.150 ± 0.053	−0.048
	VP + 5Gl	8:2	5.41 ± 0.00	5.39 ± 0.01	−0.02	610.5 ± 0.7	609.0 ± 0.0	−1.5	0.181 ± 0.003	0.180 ± 0.012	−0.001
		6:4	5.49 ± 0.00	5.48 ± 0.00	−0.01	850.5 ± 0.7	843.0 ± 5.7	−7.5	0.247 ± 0.006	0.165 ± 0.006	−0.081
		5:5	5.51 ± 0.01	5.51 ± 0.00	0.00	985 ± 2.8	980.5 ± 2.1	−4.5	0.207 ± 0.014	0.138 ± 0.006	−0.069
Lipoflex special	VP + NS	8:2	5.13 ± 0.01	5.20 ± 0.00	0.07	498.5 ± 3.5	509.0 ± 1.4	10.5	0.330 ± 0.044	0.233 ± 0.015	−0.097
		6:4	5.39 ± 0.01	5.41 ± 0.01	0.02	818 ± 2.8	817.0 ± 5.7	−1.0	0.268 ± 0.002	0.28 ± 0.045	0.012
		5:5	5.44 ± 0.00	5.45 ± 0.00	0.01	936.5 ± 2.1	938.5 ± 3.5	2.0	0.436 ± 0.037	0.393 ± 0.002	−0.043
	VP + 5Gl	8:2	5.33 ± 0.00	5.36 ± 0.01	0.03	549.5 ± 3.5	554.5 ± 3.5	5.0	0.265 ± 0.019	0.231 ± 0.005	−0.034
		6:4	5.46 ± 0.01	5.46 ± 0.01	0.01	855 ± 1.4	856.0 ± 2.8	1.0	0.372 ± 0.004	0.334 ± 0.001	−0.038
		5:5	5.48 ± 0.00	5.49 ± 0.00	0.01	1003.5 ± 2.1	1018.5 ± 10.6	15.0	0.310 ± 0.006	0.267 ± 0.034	−0.043
Kabiven	VP + NS	7:3	5.35 ± 0.01	5.36 ± 0.00	0.01	510.5 ± 3.5	522.5 ± 2.1	12.0	0.245 ± 0.002	0.207 ± 0.004	−0.038
		6:4	5.40 ± 0.01	5.41 ± 0.00	0.01	583.5 ± 3.5	594.5 ± 0.7	11.0	0.249 ± 0.006	0.263 ± 0.008	0.014
		5:5	5.46 ± 0.01	5.45 ± 0.00	−0.01	666.5 ± 0.7	679.0 ± 1.4	12.5	0.252 ± 0.001	0.270 ± 0.001	0.018
	VP + 5Gl	7:3	5.39 ± 0.00	5.38 ± 0.00	−0.01	510.5 ± 0.7	522.5 ± 0.7	12.0	0.203 ± 0.007	0.194 ± 0.005	−0.009
		6:4	5.44 ± 0.00	5.45 ± 0.00	0.01	604 ± 0.0	614.5 ± 0.7	10.5	0.241 ± 0.004	0.252 ± 0.001	0.010
		5:5	5.48 ± 0.00	5.48 ± 0.01	0.00	699 ± 0.0	704.0 ± 1.4	5.0	0.259 ± 0.002	0.276 ± 0.007	0.017
SmofKabiven	VP + NS	7:3	5.38 ± 0.01	5.38 ± 0.01	0.01	644.5 ± 0.7	647 ± 1.4	2.5	1.723 ± 0.006	1.520 ± 0.000	−0.203
		5:5	5.45 ± 0.00	5.46 ± 0.02	0.01	928 ± 2.8	928.5 ± 2.1	0.5	1.347 ± 0.147	1.183 ± 0.006	−0.163
	VP + 5Gl	7:3	5.41 ± 0.01	5.45 ± 0.01	0.04	642 ± 7.1	638.5 ± 2.1	−3.5	0.182 ± 0.002	0.169 ± 0.001	−0.012
		5:5	5.47 ± 0.00	5.48 ± 0.00	0.01	927 ± 0.0	926.5 ± 4.9	−0.5	0.247 ± 0.002	0.224 ± 0.003	−0.022
Kabiven Peripheral	VP + NS	7:3	5.28 ± 0.00	5.30 ± 0.00	0.02	471.5 ± 71.4	478 ± 69.3	6.5	0.215 ± 0.002	0.209 ± 0.019	−0.006
		5:5	5.45 ± 0.00	5.47 ± 0.01	0.02	516.5 ± 3.5	522.5 ± 0.7	6.0	0.225 ± 0.001	0.220 ± 0.006	−0.006
		3:7	5.54 ± 0.00	5.56 ± 0.00	0.02	620 ± 0.0	628.0 ± 0.0	8.0	0.221 ± 0.001	0.213 ± 0.012	−0.008
	VP + 5Gl	7:3	5.36 ± 0.00	5.35 ± 0.00	−0.01	433.5 ± 0.7	439.5 ± 2.1	6.0	0.154 ± 0.002	0.153 ± 0.004	−0.001
		5:5	5.48 ± 0.00	5.48 ± 0.00	0.00	530.5 ± 0.7	537.5 ± 0.7	7.0	0.178 ± 0.002	0.221 ± 0.010	0.044
		3:7	5.55 ± 0.00	5.56 ± 0.00	0.01	635.5 ± 2.1	642.5 ± 0.7	7.0	0.424 ± 0.012	0.539 ± 0.005	0.115
Lipoflex peri	VP + NS	7:3	5.24 ± 0.01	5.26 ± 0.00	0.02	455.5 ± 0.7	458.5 ± 0.7	3.0	1.187 ± 0.012	1.083 ± 0.006	−0.103
		6:4	5.32 ± 0.01	5.32 ± 0.01	0.00	508.5 ± 7.8	517.0 ± 0.0	8.5	1.020 ± 0.010	1.057 ± 0.021	0.037
		5:5	5.38 ± 0.00	5.83 ± 0.01	0.00	576 ± 2.8	577.0 ± 2.8	1.0	0.860 ± 0.001	0.835 ± 0.001	−0.025
	VP + 5Gl	7:3	5.31 ± 0.00	5.31 ± 0.01	0.00	467.5 ± 2.1	472.5 ± 0.7	5.0	0.486 ± 0.048	0.354 ± 0.001	−0.132
		6:4	5.35 ± 0.00	5.34 ± 0.00	−0.01	528.5 ± 0.7	530.0 ± 1.4	1.5	0.415 ± 0.025	0.52 ± 0.018	0.105
		5:5	5.39 ± 0.00	5.38 ± 0.00	−0.01	582 ± 4.2	587.0 ± 1.4	5.0	0.330 ± 0.002	0.362 ± 0.006	0.032
Omega flex peri	VP + NS	7:3	5.28 ± 0.00	5.30 ± 0.00	0.02	472 ± 2.8	473.0 ± 2.8	1.0	1.797 ± 0.064	1.543 ± 0.006	−0.253
		6:4	5.34 ± 0.01	5.36 ± 0.00	0.02	529.5 ± 0.7	529.5 ± 3.5	0.0	2.227 ± 0.012	2.063 ± 0.050	−0.163
		5:5	5.38 ± 0.00	5.40 ± 0.00	0.02	589.5 ± 0.7	582.5 ± 4.9	−7.0	2.670 ± 0.010	2.39 ± 0.010	−0.280
	VP + 5Gl	7:3	5.22 ± 0.00	5.21 ± 0.01	−0.01	461 ± 0.0	455.5 ± 0.7	−5.5	1.887 ± 0.035	1.553 ± 0.006	−0.333
		6:4	5.25 ± 0.00	5.31 ± 0.00	0.06	520 ± 0.0	520.5 ± 2.1	0.5	2.367 ± 0.035	2.117 ± 0.006	−0.250
		5:5	5.31 ± 0.00	5.37 ± 0.00	0.06	572.5 ± 3.5	580.0 ± 4.2	7.5	2.770 ± 0.010	2.440 ± 0.000	−0.330
Olimel Peri	VP + NS	7:3	6.01 ± 0.02	6.07 ± 0.01	0.06	445 ± 0.0	445.5 ± 0.7	0.5	0.275 ± 0.012	0.200 ± 0.002	−0.075
		6:4	6.13 ± 0.01	6.16 ± 0.00	0.03	493 ± 1.4	494.5 ± 0.7	1.5	0.428 ± 0.020	0.289 ± 0.003	−0.139
		5:5	6.23 ± 0.00	6.24 ± 0.00	0.01	553 ± 0.0	551.5 ± 2.1	−1.5	0.457 ± 0.046	0.227 ± 0.002	−0.230
	VP + 5Gl	7:3	6.05 ± 0.01	6.07 ± 0.01	0.02	440.5 ± 2.1	440.0 ± 0.0	−0.5	0.321 ± 0.003	0.220 ± 0.021	−0.101
		6:4	6.20 ± 0.00	6.21 ± 0.00	0.01	502.5 ± 0.7	503.0 ± 1.4	0.5	0.285 ± 0.004	0.246 ± 0.014	−0.039
		5:5	6.26 ± 0.00	6.26 ± 0.01	0.00	549 ± 1.4	550.5 ± 0.7	1.5	0.215 ± 0.005	0.186 ± 0.014	−0.030

X ± SD—average ± standard deviation; Δ—Δ (t_4h_ − t_0h_)—difference between the value from the second endpoint (t = 4 h) and the first one (t = 0 h); Δ%—(RSDt_4h_ − RSDt_0h_)—difference between the relative standard deviation value from the second endpoint (t = 4 h) and the first one (t = 0 h); VP—vinpocetine, NS—normal saline, 0.9% sodium chloride solution; 5Gl—5% glucose solutions.

**Table 4:** Comparison of compatibility results.

PNA	Drug Solution	VP:PNA Ratio	Measurements						
			pH	Osmolality	Turbidity	ZP	PDI	MDD	PFAT5
Omegaflex special	VP + NS	8:2	C	C	C	C	C	C	I
		6:4	C	C	C	C	C	C	C
		5:5	C	C	C	C	C	C	C
	VP + 5Gl	8:2	C	C	C	C	C	I	I
		6:4	C	C	C	C	C	C	C
		5:5	C	C	C	C	C	C	C
Lipoflex special	VP + NS	8:2	C	C	C	C	C	I	I
		6:4	C	C	C	C	C	I	I
		5:5	C	C	C	C	C	I	I
	VP + 5Gl	8:2	C	C	C	C	C	I	I
		6:4	C	C	C	C	C	I	I
		5:5	C	C	C	C	C	C	I
Kabiven	VP + NS	7:3	C	C	C	C	C	I	I
		6:4	C	C	C	C	C	C	C
		5:5	C	C	C	C	C	C	C
	VP + 5Gl	7:3	C	C	C	C	C	I	I
		6:4	C	C	C	C	C	I	I
		5:5	C	C	C	C	C	I	I
SmofKabiven	VP + NS	7:3	C	C	C	C	C	I	I
		5:5	C	C	C	C	C	C	C
	VP + 5Gl	7:3	C	C	C	C	C	C	C
		5:5	C	C	C	C	C	I	I
Kabiven Peripheral	VP + NS	7:3	C	C	C	C	C	I	I
		5:5	C	C	C	C	C	I	I
		3:7	C	C	C	C	C	C	C
	VP + 5Gl	7:3	C	C	C	C	C	I	I
		5:5	C	C	C	C	C	I	I
		3:7	C	C	C	C	C	C	C
Lipoflex peri	VP + NS	7:3	C	C	C	C	C	C	I
		6:4	C	C	C	C	C	C	C
		5:5	C	C	C	C	C	C	C
	VP + 5Gl	7:3	C	C	C	C	C	I	I
		6:4	C	C	C	C	C	I	I
		5:5	C	C	C	C	C	C	C
Omegaflex peri	VP + NS	7:3	C	C	C	C	C	I	I
		6:4	C	C	C	C	C	C	I
		5:5	C	C	C	C	C	C	I
	VP + 5Gl	7:3	C	C	C	C	C	I	I
		6:4	C	C	C	C	C	C	C
		5:5	C	C	C	C	C	C	C
Olimel Peri	VP + NS	7:3	C	C	C	C	C	C	I
		6:4	C	C	C	C	C	C	I
		5:5	C	C	C	C	C	C	C
	VP + 5Gl	7:3	C	C	C	C	C	I	I
		6:4	C	C	C	C	C	C	I
		5:5	C	C	C	C	C	C	C

VP—vinpocetine, NS—normal saline, 0.9% sodium chloride solution; 5Gl—5% glucose solutions, ZP—zeta potential, PDI—polydispersity index, MDD—particle size as mean droplet diameter, PFAT5—percentage of lipids residing in globules larger than 5 µm, C—compatible, I—incompatible.

## Data Availability

The data presented in this study are available upon request from the authors.
